# Medicine

**Published:** 1908-09

**Authors:** Carey Coombs


### Progress of the Medical Sciences.

### MEDICINE.

**Alterations in the rhythm of the heart.**—Thanks to the work of Wenckebach, Mackenzie, and others, it is becoming possible to distinguish a number of types of cardiac arrhythmia, and to attach to each its proper meaning. These are reviewed and arranged in papers by Mackenzie[1] and Norris.[2] Underlying the whole subject are certain essential facts.

[1] *Quart. J. Med.*, 1908, i. 39, 131, 481.
[2] *Am. J. M. Sc.*, 1908, cxxxvi. 46.

1. In the human heart are relics of the primitive cardiac tube. These are (*a*) the sino-auricular node, placed near the opening of the superior vena cava into the right auricle; (*b*) the auriculo-ventricular node, lying in the auricular wall near the mouth of the coronary sinus; and (*c*) the auriculo-ventricular bundle passing from the node (*b*) into the ventricular walls. So far specialised fibres joining (*a*) with (*b*) through the auricular walls have not been discovered.

2. Each cardiac contraction begins with a stimulus, almost certainly intrinsic, exciting the heart first at the sino-auricular node. From this point the stimulus passes through the heart along the path of the primitive tube, from node (*a*) to node (*b*), and thence into the ventricles by bundle (*c*).

3. Cardiac muscle while contracting is refractory to further stimuli; these can only excite further contractions when they fall upon partly or wholly relaxed muscle.

The types of irregularity now to be described have been separated very largely by the study of tracings of the jugular pulse, taken by the use of some form of polygraph. Mackenzie's "ink polygraph" is the best for ordinary use.

(*a*) *Juvenile arrhythmia.*—This is only an exaggeration of the normal inspiratory increase and expiratory decrease of the pulse rate. It is seen in young people, neurasthenics, and those suffering from acute fevers. Such irregularities are common after diphtheria and scarlet fever, and it is important to realise their origin in nervous instability rather than in cardiac disease.

(*b*) *Extra-systoles.*—These are superfluous cardiac contractions due to abnormal stimulations of varying parts of the primitive tube. Mackenzie divides them into three classes—auricular, ventricular and auriculo-ventricular. Auricular extra-systole is caused by stimuli arising in those fibres (as yet not demonstrated) which run from node (*a*) to node (*b*); such stimuli will pass over to the ventricular wall, and, as in normal systole, the auricular contraction is followed, after an interval, by that of the ventricle. Ventricular extra-systole is due to stimulation of the auriculo-ventricular bundle in the ventricular side of the auriculo-ventricular node. This causes an abnormal ventricular systole, without any corresponding auricular contraction. Auriculo-ventricular extra-systole is an abnormal contraction of auricle and ventricle simultaneously, due to stimuli applied to the auriculo-ventricular node, and travelling thence both ways—back to the auricle and forward to the ventricle. This might be called an "occasional nodal rhythm." In one type of extra-systole, then, the auricle does not contract at all, in the auricular type it contracts as usual before the ventricle; while in the third type both auricle and ventricle contract together.

(*c*) "*Perpetual*" *arrhythmia.*—This is the irregularity met with in the serious heart failure of mitral obstruction and arteriosclerosis. Mackenzie believes it to be due to interruption of the passage of stimuli through the auricular walls by over-distension of the auricle ; stimuli arise no longer at the usual spot, the sino-auricular node, but instead at the auriculo-ventricular node. Thus a "persistent nodal rhythm" is caused, characterised by simultaneous contraction of auricle and ventricle, as polygraph records show.

(*d*) *Heart-block.*—Recent papers on the Stokes-Adams syndrome are making us familiar with this class of arrhythmia. It occurs also in a less definite form—that of occasional dropping of beats—in arterio-sclerotics and persons recovering from influenza and other infections attacking the myocardium. In these conditions some lesion of the auriculo-ventricular bundle hinders the regular passage of stimuli from auricle to ventricle ; the auricle beats regularly, but the ventricle loses a certain number of stimuli, and therefore of beats also. The radial pulse is therefore "slower" than the jugular.

(*e*) *Pulsus alternans.*—This, a rare form, is seen in cases of myocardial failure. There is a regular and persistent alternation of large and small pulse waves at the wrist. Wenckebach says that it begins with a systole slightly more effective than usual, for some reason ; this would make no difference to the rhythm of a normal heart, but in a weakened ventricle it means a lengthening of the interval occupied by that particular beat. At the time, therefore, that the next stimulus comes down from the auricle to the ventricle it finds the latter still imperfectly relaxed, and therefore partially "refractory," so that the contraction provoked by this stimulus is weak and short. Being short, it gives the heart an unduly long rest, so that the ventricle is now able to answer the next stimulus by an abnormally powerful systole. This means another lingering diastole, another small systole, and so on *ad infinitum.*

(*f*) *Paroxysmal tachycardia.*—This is a "functional" disorder of rhythm, that is to say, it is not connected with any grave morbid change in the structure of the heart. Mackenzie believes it to be a periodic discharge of auriculo-ventricular extra-systoles ; but Hirschfelder[1] and others, much impressed by the fact that in the paroxysms the heart beats exactly twice (less often exactly three or four times) as often as its normal rate, say that in such hearts there is always a great frequency of stimuli at the sino-auricular node, that between the attacks half of the stimuli (or in less common cases two-thirds or three-quarters) are stopped before reaching the ventricle, while in the attacks this hindrance is removed, and all the stimuli are allowed to pass through the ventricle. There is some experimental evidence in favour of this theory.

It is, of course, impossible to generalise with regard to the

[1] *Johns Hopkins Hosp. Bull.*, 1906, xvii. 337.

prognosis and treatment of conditions differing so widely in origin. Clearly it is useless to take arrhythmia into account in any case of cardiac disease without forming a definite opinion as to the nature and cause of the irregularity.

**Congenital idiopathic dilatation of the colon (Hirschsprung's disease).**—A paper by Finney,[1] reporting a fresh case and summarising two hundred other contributions, gives a full account of this uncommon disease.

*Ætiology.*—In most cases the disease dates from birth or soon after; in a very few it seems to arise *de novo* in adult life. It much more commonly affects males. Heredity is of no importance. The actual origin of the disease is a matter of controversy, and many theories have been advanced. It has been ascribed to abnormal length of the meso-sigmoid, an abnormally long and looped colon, colitis causing gaseous distension, a developmental weakness of the colonic muscle, some obstruction in the lower reaches of the colon, spasm of the sphincter ani, a neuro-muscular defect of one segment of the colon, valvular formation in the mucosa, and so on. In Finney's case certain appearances suggested a kind of giantism, with lymphangiectasy (analogous to macroglossia) as the true explanation. Out of all these theories no single one can be held to explain all cases. It seems, however, that it is right to look upon the disease as congenital in a majority of cases, and to consider the "obstruction" factor as negligible. One point of importance is not remarked upon, namely the occasional association of dilatation of the colon with other congenital defects. In a case I saw, congenital heart disease, congenital cataract, enlargement of the colon, and Mongolism were all present in the same patient.

*Pathology.*—Nearly always it is the sigmoid loop that is chiefly enlarged. The dilated bowel may hold as much as 40 lb. of fæces, and a diameter of six to eight inches has been recorded. The wall of the colon is very thick, owing to muscular hypertrophy, and the meso-colon is thickened by the presence of many blood-vessels and lymph-channels. The mucosa is pigmented, and sometimes ulcerated; the surface epithelium is usually degenerate, and its deeper crypts relatively deficient in goblet-cells. There is general cellular infiltration of the sub-mucosa. The circular muscle is the more hypertrophied layer.

*Symptoms.*—There are two cardinal symptoms—constipation and abdominal enlargement. The bowels are extraordinarily difficult to move, even by the most forcible aperients and enemata. Patients have been known to go for three months without an action. When a motion is passed it is naturally very bulky, and pasty in consistency. The belly may be so enlarged that the skin

[1] *Sur., Gynec. and Obst.*, June, 1908.

is shiny and the recti parted. It is chiefly above the umbilicus that the enlargement is seen. Often peristaltic waves are visible, crossing the upper abdomen from right to left. At such times the colon is hard, but at others it is soft, and "splashes" when the fingers are thrust down into it.

Other symptoms that are met with belong rightly to two classes —those caused mechanically, and those which are toxic in origin. Among the latter may be mentioned mental apathy—a common feature— and tetany, which has been recorded.

*Diagnosis.*—A fæcal mass has before now been considered malignant until it has vanished under the influence of enemata; and the abdominal enlargement has before now led to a diagnosis of peritoneal tuberculosis.

*Prognosis.*—The disease cannot truly be cured. It does not often kill, except by allowing intercurrent affections to find an unresisting victim.

*Treatment.*—Aperients and enemata, with diet and abdominal massage, will often make the patient's state readily endurable, but in bad cases the bowels will become more and more difficult to move. Finney is led to uphold surgical treatment by the comparative success of such measures as judged from a tabulation of recorded cases. In his own case, a three-stage operation (colostomy above the dilatation, lateral anastomosis of colon to sigmoid, "side-tracking" the dilated portion, and finally colectomy) was highly successful.

**The part played by rheumatism in causing disease of blood-vessels.**—A recent paper by Roch and Burnand, of Geneva,[1] dealing chiefly with peripheral rheumatic arteritis, also touches on other forms of blood-vessel disease due to acute rheumatism.

*Rheumatic phlebitis* has been described by so many writers, that we may consider its claims to recognition fully established. In eighty-eight autopsies of cases of rheumatic heart disease fatal before 16 in the Bristol hospitals' records, there was one case of phlebitis. In this case it was, as is usual, an inflammation of the jugular veins.

*Rheumatic aortitis* has also been written upon by many distinguished observers in this country and in France. Small patches of subendothelial inflammation are to be seen in nearly every case of fatal acute rheumatic carditis in the first two or three inches of the aorta. Further than this, however, it comes into clinical importance as an early stage of a process which may end in the formation of an aneurysm. This subject was dealt with some years ago by Sir R. Douglas Powell, while quite lately it has occupied the attention of other writers. Ralli,[2] in a graduation

[1] *Semaine Méd.*, March 25th, 1908.

[2] Inaugural Dissertation, Bukarest, 1907 (*Zentralbl. f. innere Med.*, 1907, xxviii. 1239).

thesis, speaks of rheumatism as a very important factor in the causation of aortitis and aneurysm in children. Feytaud[1] says that many cases of aortic aneurysm are met with in patients who have never had syphilis, but in whom a rheumatic attack is not long past. Such patients are usually young, and give a history of often-repeated bouts of rheumatism, each of which seems to add to the aortic enlargement. This usually arises from the ascending portion, and spreads forwards and to the right.

*Coronary arteritis* due to rheumatism has been several times discovered, and the relation between the coronary vessels and the interstitial lesions of rheumatic carditis is always very close. In a coronary arteriole in one of my own sections, the media was occupied and the lumen almost filled by one of the "submiliary nodules" characterising rheumatism of the heart wall.

*Peripheral arteritis of rheumatic origin.*—Of the many cases reported, Roch and Burnand consider that five only can be accepted: to these they add a sixth observed by themselves. They divide the cases into two classes. In the first of these the inflammation was apparently parietal, and attacked the media principally; the symptoms being tenderness, pain and increased pulsation at the point of disease. The other group included two cases only, those in which the inflammation led to the temporary or permanent obliteration of long segments of arteries, with abolition of the pulse and the appearance of nodose swellings along the course of the vessel. They give their reasons for believing that in these cases the lesion was arteritis, and not embolism; but their statements, though sane and convincing as far as they go, are not supported by any *post-mortem* evidence, so that it is not yet possible to consider the existence of a peripheral rheumatic arteritis (apart from embolism) as proved. It is worth noting, in conclusion, that Hanot believed acute rheumatism to be productive of arterio-sclerosis, an idea which has lately found a supporter in Fremont-Smith,[2] who assigns a prominent place to rheumatism as a cause of arterio-sclerosis in early life.

CAREY COOMBS.

[1] *Thèse de Paris*, 1906. [2] *Am. J. M. Sc.*, 1908, cxxxv. 199.

